# Global Data for Ecology and Epidemiology: A Novel Algorithm for Temporal Fourier Processing MODIS Data

**DOI:** 10.1371/journal.pone.0001408

**Published:** 2008-01-09

**Authors:** Jörn P. W. Scharlemann, David Benz, Simon I. Hay, Bethan V. Purse, Andrew J. Tatem, G. R. William Wint, David J. Rogers

**Affiliations:** 1 Spatial Ecology and Epidemiology Group, Department of Zoology, University of Oxford, Oxford, United Kingdom; 2 Malaria Public Health and Epidemiology Group, Centre for Geographic Medicine, Kenya Medical Research Institute (KEMRI), University of Oxford, Wellcome Trust Collaborative Programme, Nairobi, Kenya; University of Southampton, United Kingdom

## Abstract

**Background:**

Remotely-sensed environmental data from earth-orbiting satellites are increasingly used to model the distribution and abundance of both plant and animal species, especially those of economic or conservation importance. Time series of data from the MODerate-resolution Imaging Spectroradiometer (MODIS) sensors on-board NASA's Terra and Aqua satellites offer the potential to capture environmental thermal and vegetation seasonality, through temporal Fourier analysis, more accurately than was previously possible using the NOAA Advanced Very High Resolution Radiometer (AVHRR) sensor data. MODIS data are composited over 8- or 16-day time intervals that pose unique problems for temporal Fourier analysis. Applying standard techniques to MODIS data can introduce errors of up to 30% in the estimation of the amplitudes and phases of the Fourier harmonics.

**Methodology/Principal Findings:**

We present a novel spline-based algorithm that overcomes the processing problems of composited MODIS data. The algorithm is tested on artificial data generated using randomly selected values of both amplitudes and phases, and provides an accurate estimate of the input variables under all conditions. The algorithm was then applied to produce layers that capture the seasonality in MODIS data for the period from 2001 to 2005.

**Conclusions/Significance:**

Global temporal Fourier processed images of 1 km MODIS data for Middle Infrared Reflectance, day- and night-time Land Surface Temperature (LST), Normalised Difference Vegetation Index (NDVI), and Enhanced Vegetation Index (EVI) are presented for ecological and epidemiological applications. The finer spatial and temporal resolution, combined with the greater geolocational and spectral accuracy of the MODIS instruments, compared with previous multi-temporal data sets, mean that these data may be used with greater confidence in species' distribution modelling.

## Introduction

Environmental variables, such as temperature and vegetation greenness, are important determinants of the distributions of many species [1]. The presence or absence of a species in any area is often distinguished not only by the absolute levels of climate or vegetation values, but also by subtle differences in the seasonality of these variables [2], which can only be captured by repeated measurements over time. Such time series may be derived from ground-based meteorological records, but acquiring spatially continuous, global records of these environmental variables is only practical using remotely sensed data from Earth-orbiting satellites. Historically, the National Oceanographic and Atmospheric Administration (NOAA) series of satellites carrying the Advanced Very High Resolution Radiometer (AVHRR) have provided time series of global imagery more or less continuously since 1981 [3]–[5]. These time series have been used to produce, among others, images of Land Surface Temperature (LST) [6] and of the Normalised Difference Vegetation Index (NDVI), a correlate of vegetation productivity, biomass and climatic conditions [7].

Serial correlation among successive observations taken over a period of time reduces the statistical utility of captured imagery. Data reduction (ordination) methods are usually employed to remove these correlations and provide one or more transformed images without such correlation, which can then be used in further analyses or applications. One ordination approach commonly applied to multi-temporal imagery is principal components analysis (PCA, e.g. [8]), but explicit measures of seasonality are lost in the ordination process. PCA thus achieves data reduction at the expense of biological descriptiveness. Alternative methods that retain information about seasonality include polynomial functions [9], [10] and temporal Fourier analysis [11]–[19].

Temporal Fourier analysis (TFA) transforms a series of observations taken at intervals over a period of time into a set of (uncorrelated) sine curves, or harmonics, of different frequencies, amplitudes and phases that collectively sum to the original time series. For many multi-temporal satellite data, the most important harmonics are those that correspond to the annual, bi-annual and tri-annual cycles of seasonal changes, and these harmonics often have a clear biological interpretation [13]. Both longer period cycles (variation on inter-annual scales) and shorter period cycles (high frequency intra-annual variation) can also be identified by TFA, but tend to be less important biologically, as well as in terms of their contributions to the overall variance of the signal [13]. Thus TFA achieves data ordination in a biologically transparent way.

An additional advantage of TFA is that it can be used to smooth noisy data. Fourier analysis moves between the time and frequency domains: forward analysis produces a frequency domain representation of the original time series and inverse analysis moves from the frequency domain back to the time domain. Filtering noisy data is easier in the frequency domain because most noise is associated with high frequencies which can therefore be dropped before inversion to produce a smoothed version of the original time series. Equivalent filtering in the time domain is less straightforward, because the high frequency components are mixed in with all other frequency components and so cannot easily be separated from them. Different degrees of smoothing occur when different frequency ranges are excluded during the filtering process. Here the primary objective is not to smooth the data, but to capture their seasonality. Smoothing should be regarded as an additional advantage of the Fourier approach to capturing seasonality; an advantage that is all the more important when, for various reasons, the satellite signal is above (e.g. sun-glint) or below (e.g. cloud contamination) its correct value.

Until relatively recently, global remotely sensed time series data have been available either with low spatial resolution for long time periods (e.g. 20 years of AVHRR at 8 km resolution) or with higher resolution for a shorter time period (e.g. 4 years of AVHRR at 1.1 km resolution) [20]. These data, when temporal Fourier processed [20], have been used successfully to predict the distributions of species [13], [14], [21]–[27], diseases [2], [17], [28]–[37], endemic bird areas [38], livestock [39], and human poverty [40].

Since 2000, new time series of higher resolution (250 m to 1 km) remotely sensed data from the MODerate-resolution Imaging Spectroradiometer (MODIS) on board the NASA Terra and Aqua satellites have been made freely available to the research community [41]. The advantages of MODIS data over previously available global satellite data include greater repeat frequency with global image collection on an almost daily cycle by each satellite, and enhanced stability of both spectral and geolocational accuracy [42]–[44]. Nevertheless, the quality of MODIS images, as with AVHRR, is affected by atmospheric contamination (clouds and aerosols). MODIS images are therefore composited over 8 or 16 days using cloud-screening algorithms, shorter time intervals than the 10 day (dekads) or one month intervals over which AVHRR images were maximum value composited. Whilst the dekadal and monthly composites of AVHRR data can be analysed by standard temporal Fourier processing methods, since their mean capture dates may be assumed to be spread equally throughout the year, the 8- and 16-day MODIS data present unique problems to such algorithms, because the timing of the samples near year-end do not overlap to give these same inter-sample intervals (a strict requirement of temporal Fourier analysis).

Further problems may arise from data points with very low or “drop-out” values which occur frequently in some pixels despite compositing. These need to be treated carefully during image processing, because they do not represent earth surface conditions at the time of image capture.

Here we present a novel algorithm to deal with both the data drop-outs and the irregular timing problems of MODIS data to produce global 1 km resolution temporal Fourier processed layers that describe the seasonality in MODIS Terra NDVI, Enhanced Vegetation Index (EVI), Middle Infrared (MIR), and daytime and night-time LST for the period 2001 to 2005 inclusive.

## Materials and Methods

### Data

Time series of nominal 1 km spatial resolution MODIS data from the NASA Terra satellite were downloaded from NASA's EOS data gateway (http://edcimswww.cr.usgs.gov/pub/imswelcome/) for five complete years, January 2001 to December 2005. MODIS data are produced in the sinusoidal projection (MODLAND Sinusoidal Grid) and made available as 460 tiles covering the Earth, each tile measuring 10°×10° and consisting of 1200×1200 0.859 km^2^ (926.63 m×926.63 m) pixels. All available images per time interval (as of 8 January 2007), called granules, were acquired for 229 tiles, including all tiles between 90°N and 60°S, except for 129 oceanic tiles and 62 tiles containing small islands, for two data sets: MODIS/Terra Land Surface Temperature/Emissivity 8-day L3 Global 1 km SIN grid (MOD11A2, version 4, [45]) and MODIS/Terra Nadir BRDF-Adjusted Reflectance 16-day L3 Global 1 km SIN grid (MOD43B4, version 4, [46]). MODIS data sets are provided in Hierarchical Data Format (HDF), and were imported to ERDAS Imagine 9.0 (Leica Geosystems, Norcross, GA) and converted to ERDAS LAN format.

The MOD11A2 data set comprises 8-day composited land surface temperature (LST) for daytime (dLST) and night-time (nLST) overpasses [45]. A complete time series for each tile of the MOD11A2 data would therefore consist of 46 granules at 8-day intervals for each of five years, or 230 granules in total.

The MOD43B4 data set provides nadir Bidirectional Reflectance Distribution Function (BRDF)-adjusted reflectances for Terra MODIS spectral bands 1–7 computed with the mean solar zenith angle of each 16-day interval over which data were composited [46]. The BRDF removes directional effects of view angle and illumination, providing reflectance values as if every pixel were viewed from nadir. Pre-processing excluded pixels with unreliable BRDF corrections, identified by quality control flags provided with the data set (QC Word 2 value >10). For each pixel a MIR channel (MODIS band 7, 2105–2155 nm) was extracted and the NDVI ([near infrared (NIR)–RED]/[NIR+RED], where NIR is MODIS band 2 and RED is band 1, 841–876 nm and 620–670 nm, respectively) and the EVI (2.5*[[NIR-RED]/[NIR+6.0*RED–7.5*BLUE+1.0]], where BLUE is MODIS band 3, 459–479 nm, [43]) were calculated. The MIR band was selected as being similar to band 3 in AVHRR, which has been shown to correlate with a number of vegetative processes including forest re-growth [47]. A complete MOD43B4 time series for each tile would consist of 23 granules at 16-day intervals for each of five years, or 115 granules in total. Although finer resolution data are available for NDVI and EVI (MODIS/Terra Vegetation Indices 16-day L3 Global 250 m resolution, MOD13Q1), MIR and LST data are only available at 1 km resolution. For consistency across products and given the much greater time involved in processing higher resolution data on a global scale, 1 km resolution data were therefore used for all products.

After temporal Fourier processing (described below), outputs for all five products were mosaicked and georeferenced (parameters in Table S1). Ocean pixels in all output layers were masked using the MODLAND Digital Elevation Model (DEM) and Land/Water Mask version 4, downloaded from ftp://landsc1.nascom.nasa.gov/pub/outgoing/dem_sin_old for all 229 tiles and processed in ERDAS Imagine 9.0 and ArcInfo 9.1 (ESRI, Redlands, CA). Since the MOD11A2 data set had been masked by the version 4 land/water mask prior to production, and this mask did not match the later version 5 mask extents based on MOD43B4 reflectance data [48], the version 4 mask was used throughout. Information on inland water and ephemeral water bodies was also extracted from the MODLAND version 4 land/water mask.

### Temporal Fourier analysis

TFA describes environmental cycles, such as temperature, precipitation, and vegetation phenology, as the sum of a series of sine curves with different amplitudes and phases. A time series [x*_t_*] may be described by its Fourier series representation [49] where
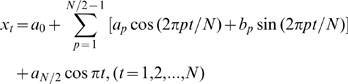
(1)with coefficients [*a*_p_, *b*_p_] defined as follows, where *x̂* is the arithmetic mean of the time series:
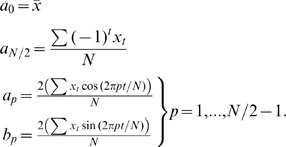
(2)


The component at a frequency ω*p* = 2π*p*/*N* is called the *p*th harmonic, and for all p ≠ *N*/2 these harmonics may be written in the equivalent form
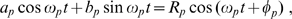
where
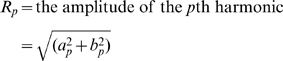
(3)and
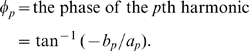



Full TFA partitions the variability of the time series into orthogonal (i.e. uncorrelated) harmonics at frequencies of 2π/*N*, 4π/*N*, 6π/*N* …, π, or periods equal to 1, ½, 1/3, … 2/*N* times the duration over which *N* observations were made. Full TFA exactly describes the original time series, because there are as many harmonic variables (*a*,*b*) as there are data points. However, in practice only a few harmonics usually contribute substantially to the overall variance, and only these need to be calculated for most purposes. For the MODIS data only the three harmonics corresponding to the annual, bi-annual and tri-annual seasonal cycles were extracted and saved.

The contribution of each of the *N*/2 harmonics to explaining the total variance has been shown by Parseval's theorem:
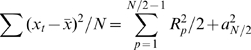
(4)where *R* ^2^ *_p_*/2 is the contribution of the *p*th harmonic.

Equation 4 shows that the Fourier harmonics are statistically orthogonal, because the total variance of the time series is described in terms of their harmonics only, and not of their co-variances. However, in contrast to the principal components of PCA, each of the harmonics of TFA has a clear interpretation in terms of intra- and inter-annual cycles of changes in their respective variables [13].

### Temporal Fourier analysis applied to artificial data

In applying temporal Fourier analysis to time series data, most standard TFA algorithms require that the data points are equally spaced in time [50]. For the extraction of annual, bi-annual and tri-annual harmonics, the data should be collected or composited at intervals that divide an integer number of times into a 365-day year, with a nominal collection date half-way through each interval. The historic AVHRR data essentially conformed to these requirements (e.g. [20]); each month was divided into 3 dekads (of, on average, just over 10 days' duration), without any difference in timing over year's end. However, MODIS data sets are provided at fixed 8- or 16-day intervals, regardless of calendar dates and always beginning at the start of each year (i.e. the first image of each calendar year always refers to the first 8 or 16 days of the year, counting from January 1^st^). Not only do these intervals not divide an integer number of times into a year, but the last interval of the previous year overlaps with the first interval in a given year (with the degree of overlap varying between leap and non-leap years).

The MODIS intervals divide into a 365-day year 45.63 or 22.81 times, for the 8- and 16-day intervals, respectively, and either 46 or 23 images per year are produced. The compositing period of the last image of each year therefore overlaps that of the first image of the following year and its nominal sampling date is taken here as exactly one sample interval after that of the previous image. Since the day counter is reset on January 1^st^ each year, this means that there is irregular timing of the images at the end of each year.

Ignoring the effects of both sample interval and unequal timing causes errors in TFA outputs using standard TFA algorithms. The precise extent of these errors was investigated by constructing artificial time series of daily data by summing annual, bi-annual and tri-annual harmonics, whose amplitudes and phases were selected at random from realistic ranges of values (0.05–1.0 for amplitudes and 0–2π for phases). The artificial time series were generated repeatedly (9900 times) and then sampled on exactly the same dates as the mid-points of the MODIS imagery, i.e. artificial MODIS data with known amplitudes and phases were generated. These artificial MODIS time series were then analysed using standard TFA methods (i.e. assuming equal spacing of the imagery) and the outputs (calculated amplitude and phases) were compared, using least-squares linear regression, with the input amplitudes and phases. Analyses were performed using the TFA module in IDRISI Andes (Clark Labs, Worcester, MA) and customised TFA algorithms developed by the authors. There were sufficient discrepancies between the input and output amplitudes and phases (see Results) to merit the development of an alternative approach to eliminate them.

### Temporal Fourier analysis of MODIS data

**Figure 1:**
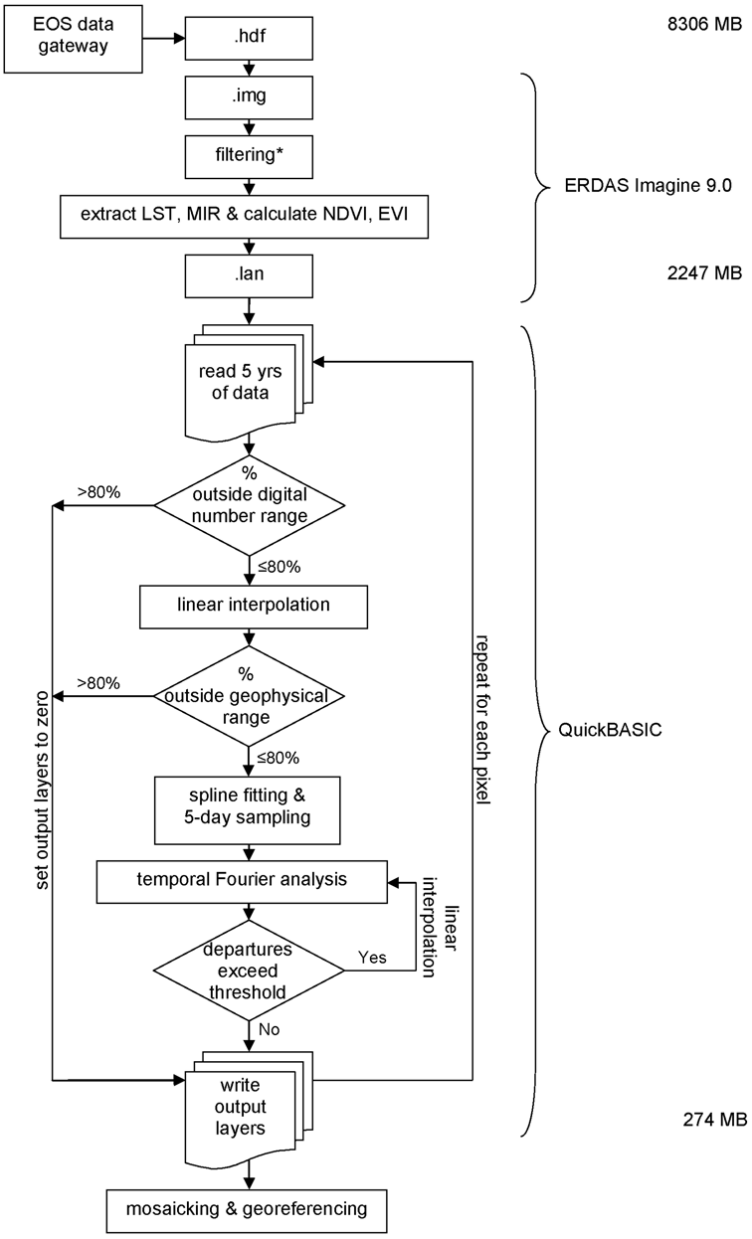
Processing chain of MODIS temporal Fourier analysis. Data storage requirements for each product (in MB) and software used are indicated on the right.

Three problems need to be addressed when TFA processing MODIS data: data drop-out and the two problems of timing in the MODIS data sets (see above). The following processing chain, developed to overcome all three problems, was implemented in QuickBASIC 4.0 (Microsoft, Redmond, WA) and applied to each pixel (Figure 1).

*i)* For each pixel, the full time series was extracted from the LAN files and examined for obvious drop-out values and for missing granules. Drop-out values were identified by low (0) or very high (>32500) digital numbers and removed from the series. If more than 80% of any time series was classified as drop-outs, the TFA output layers were set to zero, indicating that no TFA was possible for this pixel. If 80% or fewer of the values were classified as drop-outs, then the algorithm moved to the next step. This rather high value of 80% was selected so as not to exclude far more pixels–especially in higher latitudes–thus preventing the production of TFA imagery for these regions.
*ii)* The digital numbers of the time series were converted to geophysical values using the scales and offsets of the relevant MODIS product. If these geophysical values were outside a wide range considered reasonable for each product (Table 1), the value for that particular granule was deemed unreliable. Again, if more than 80% of the values in a time series were classified as unreliable, the TFA output layers were set to zero.
*iii)* A pixel passing the first two steps of processing thus potentially contained a suitable time series, although many pixels still had numerous drop-out values. At this stage, missing values were linearly interpolated from adjacent sample dates with acceptable geophysical values. Linear interpolation often spanned multiple missing values, and occasionally was also necessary at the end of the time series, where data wrap-around to the start of the time series was assumed. Linear interpolation was adopted as the simplest gap-filling approach; more complicated methods would have been more time consuming and would involve additional arbitrary decisions (e.g. the number of data points either side of any gap to include in any local, non-linear gap-filling algorithm). In general TFA is itself a non-linear gap-filling routine (from the viewpoint of the entire time series), so that the details of local gap filling are probably immaterial.
*iv)* After linear interpolation of the missing values in the time series, cubic splines were fitted to the time series. These splines not only passed through all the values in the time series, but also joined them smoothly. The spline fits could therefore be calculated at any point in the time series and at user-specified intervals, e.g. daily, 5-day, etc., as required. By sampling the spline fits at the mid points of 5-day intervals throughout the year (beginning at day 2.5), a new time series was produced with 73 5-day interval values per year, making the series suitable for subsequent standard TFA processing.
*v)* The Fourier fit to the time series was produced by summing the mean and annual, bi- and tri-annual cycles (only), and this was compared to the spline-interpolated data. Where the spline-interpolated data departed (both positively and negatively) from the Fourier fit by more than a user-specified threshold (see Table 1) the data were again considered unreliable, removed and linearly interpolated from adjacent, reliable values in the spline-fitted series. TFA was applied again to this new series, examined for departures as before, interpolated if necessary, and Fourier processed again. Re-processing continued until no departures from the current fit exceeded the threshold or until 20 iterations were completed.

**Table 1:** Number of granules used, and permitted geophysical values during temporal Fourier processing.

Product	No. of granules	minimum geophysical value	maximum geophysical value	maximum departure
dLST	52144	220°K	390°K	5°K
nLST	52144	220°K	390°K	5°K
MIR†	25697	0.0001	1	0.1
NDVI†	25697	−0.2	1	0.2
EVI†	25697	−0.2	1	0.2

Column (i) lists the 5 products processed, i.e. daytime Land Surface Temperature (dLST), night-time LST (nLST), Middle Infrared Reflectance (MIR), Normalized Difference Vegetation Index (NDVI) and Enhanced Vegetation Index (EVI).Column (ii) contains the number of granules available from 2001–2005 for 229 MODIS tiles. Column (iii) and (iv) give the permitted minimum and maximum geophysical values during temporal Fourier processing, and column (v) the maximum permitted departure of the spline interpolated geophysical value from the fitted Fourier value.

† MIR, NDVI and EVI are dimensionless ratios.

The characteristics of these final Fourier fits were saved as a series of output layers, including the mean (a0), amplitudes (a1–a3), and phases (p1–p3), the minimum (mn), maximum (mx) and variance (vr), and the proportions of the signal variance captured by the annual, bi-annual and tri-annual cycles (d1,d2, and d3, respectively) and by all cycles combined (da). The percentage data loss at steps *i*) and *ii*), as well as the percentage of departures of the spline-fitted values from the initially fitted Fourier series (step *v*) were also stored in separate layers (e1–e3), to aid the quality assessment of output layers.

## Results

### Data

**Figure 2:**
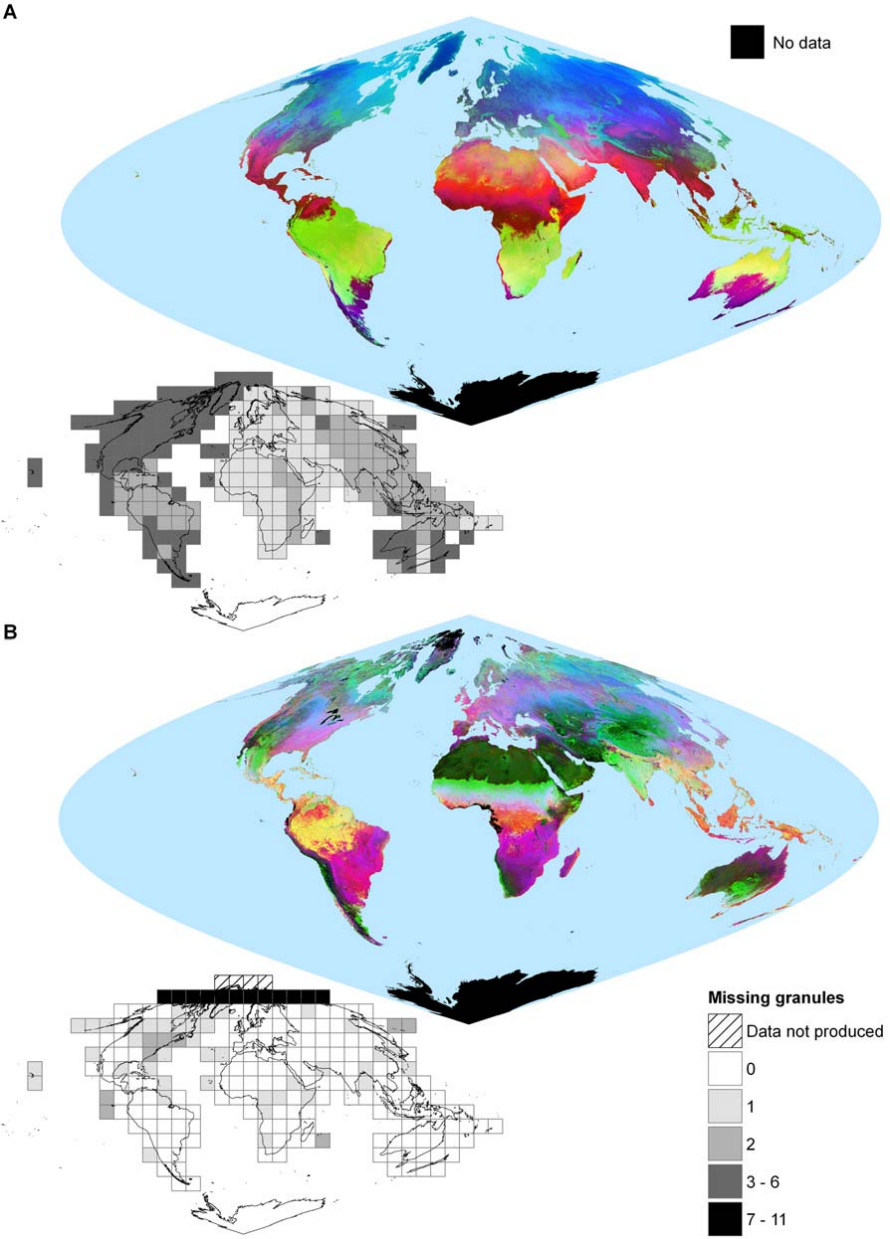
Temporal Fourier analysis of global (a) daytime Land Surface Temperature (dLST) and (b) Enhanced Vegetation Index (EVI). The analyses were based on the period 2001–2005 using 230 images at 8-day intervals for dLST (a) and 115 images at 16-day intervals for EVI (b), both resampled at 5-day intervals after cubic spline interpolation. Data are displayed as three-channel colour composites with the mean, phase and amplitude of the annual harmonic in the red, green and blue channel, respectively. For display purposes, values in each band were stretched across the full range of intensities within the image processing system using histogram equalization. The insets show the 229 MODLAND tiles that were processed, indicating the number of missing granules in each. Data are displayed in MODIS sinusoidal projection.

52144 granules were acquired for the MOD11A2 data set, and 25697 for MOD43B4, representing 99.0% and 99.3% of the potential granules respectively (note MOD43B4 data were not produced for 4 tiles north of 80°N). The median number of missing granules per tile was 2 (range 1–6 granules) for MOD11A2 and 0 (0–11) for MOD43B4 (insets in Figure 2). The larger number of missing granules in the MOD11A2 data set was due to a power supply anomaly in the sensor from 16 June to 1 July 2001, preventing data collection during one or two 8-day intervals (Julian day 169 and 177) for every tile. Several granules were missing during the winter months for tiles located at high latitudes, as there was insufficient sunlight to reflect back to the satellite. Other sensor problems and failed storage tapes at the data distribution centre accounted for further missing granules.

### Temporal Fourier analysis applied to artificial data

**Figure 3:**
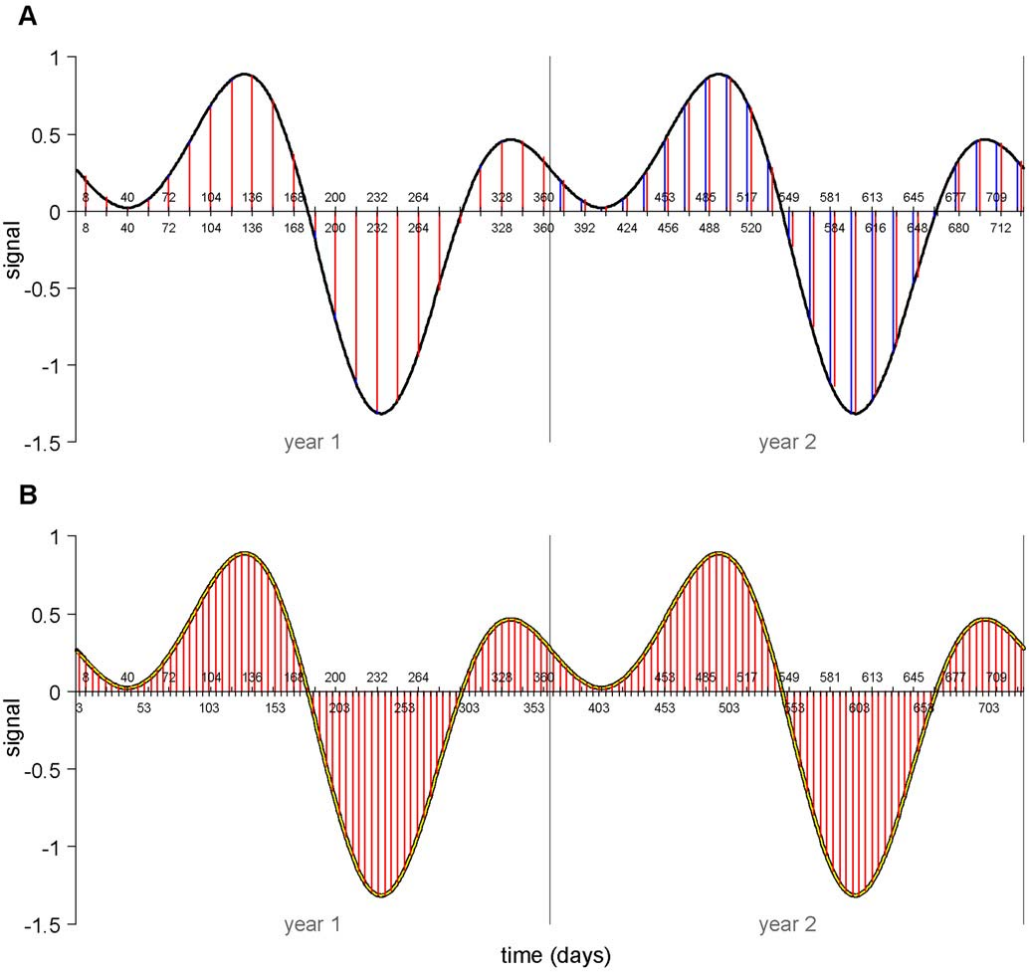
Examples of temporal Fourier processed artificially generated MODIS data for two years using (a) a standard TFA algorithm and (b) a standard TFA algorithm applied to spline-interpolated data. The signal (black line) is a daily time series artificially generated by summing annual, bi-annual and tri-annual cycles of known, randomly chosen amplitudes and phases. The ‘satellite sample’ (blue vertical lines) samples this signal at the MODIS 16-day interval and on the MODIS mid-sample date, which gives unequal intervals spanning each year end (upper tick marks on x-axis). The Fourier fit (red vertical lines) is the fit to the satellite signal that ignores this beginning/end of year anomaly and thus assumes a constant interval throughout, corresponding to the 23 images per year of the satellite sample (lower tick marks on x-axis). In (b) the daily spline fit (yellow line) is the cubic spline fit to these irregular satellite sample data. The Fourier fit (red vertical lines) is the TFA fit to the spline fit resampled every 5 days (lower tick marks on x-axis). Notice that there are no end-of-year anomalies here, resulting in a more accurate estimate of the harmonics used to generate the signal. The end of year anomaly is also present in MODIS data that run for only one year and hence affects TFA outputs in the same way, but is more clearly demonstrated visually in multi-year data.

An example time series of the artificial MODIS data subjected to standard TFA is shown in Figure 3a. The slippage between the observed and fitted values at the end of the first year is visible. TFA has assumed an equal interval throughout the 2-year time period of these artificial data, and predicts the signal at these intervals throughout. This assumption affected the estimated values of amplitudes and phases, so that the fitted values (in red) did not capture the signal satisfactorily (obvious at the very first fitted value, but noticeable throughout). This would also occur if only one year's worth of MODIS-type data were analysed by TFA since the method assumes the time series continues, as measured, forever. Application of the new TFA algorithm to the same time series is shown in Figure 3b. The 5-day spline interpolated time series, itself an accurate representation of the input time series, was described very accurately by TFA, and provides more accurate estimates of the input values of mean, amplitudes and phases.

**Figure 4:**
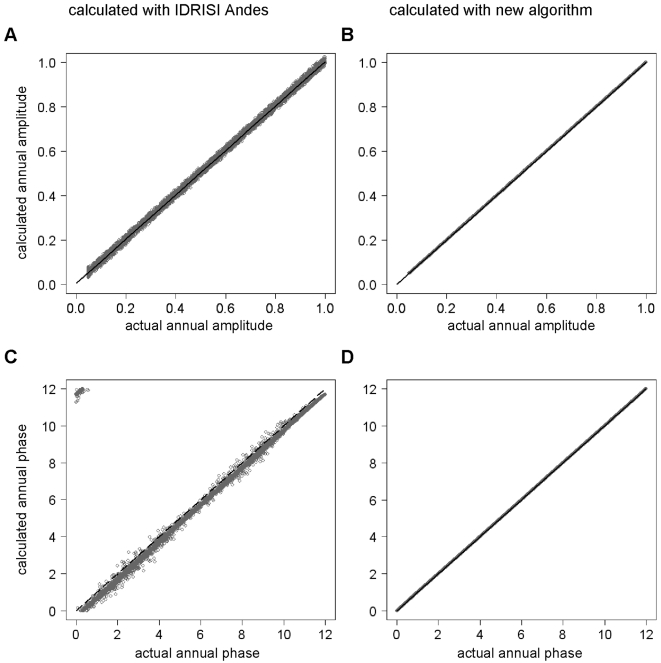
Comparisons between actual and calculated annual amplitudes (a,b) and phases (c,d) for artificially generated MODIS data. Amplitudes and phases were either calculated by standard temporal Fourier analysis (TFA) using the TFA module in IDRISI Andes (a,c) or by using the TFA algorithm described here (b,d). Lines represent least-squares regression slopes with the following equations: (a) 0.00393+0.99928*x*, *F*_1,9898_  = 1.159e+07, R^2^ = 0.9991; (b) −9.398e-06+1.0*x*, *F*_1,9898_ = 2.367e+10, *R* ^2^ = 1.0; and (d) 7.140e-05+1.0*x*, *F*_1,9898_ = 38.9e+10, *R* ^2^ = 1.0, all highly statistically significant (*P*<0.001). IDRISI Andes provides phase estimates in radians which are here re-expressed in terms comparable to the input data. Because the points in the upper left part of (c) strongly influence regression calculations, no regression was fitted to these data. Instead, the line of equality (*x* = *y*) is shown in (c) to aid visual comparison of the results.

Figure 4 highlights errors in the calculation of both annual amplitudes (Figure 4a) and annual phases (Figure 4b), found when many artificially generated MODIS time series, such as that shown in Figure 3, were subjected to standard TFA and the input amplitudes and phases compared with the TFA-calculated amplitudes and phases. These errors, both positive and negative, appeared to be approximately constant in their absolute values across the range of input values used, with the consequence that the proportional errors were very much greater at smaller input values of amplitude and phase. Thus Fig. 3a shows that using standard TFA, the annual amplitude may be estimated with an error of as little as about ±2% at high amplitude values, but as high as ±20% at low values. The equivalent values for phase are ±3% and ±30%, respectively.

Spline interpolation and resampling, followed by standard TFA, overcame the two problems of the irregular sampling dates of the raw MODIS data over all ranges of amplitudes and phases tested (Figure 4b,d).

### Temporal Fourier analysis of MODIS data

**Figure 5:**
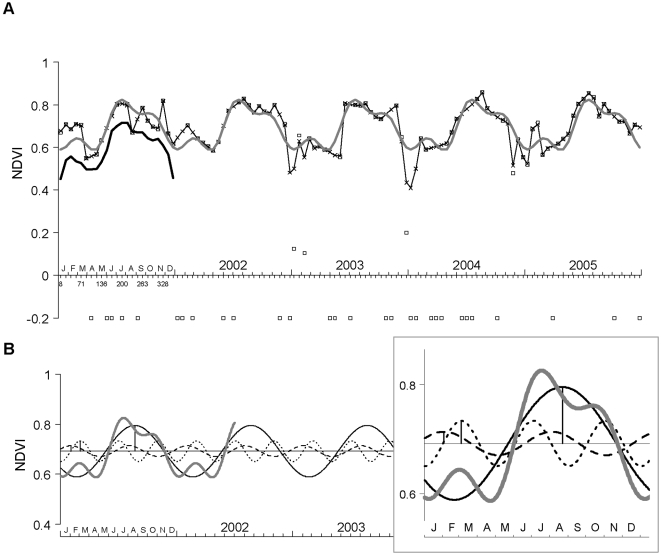
An example of temporal Fourier analysis. TFA of NDVI from a pixel in the Yorkshire Dales, England (2°W, 54°N) for the years 2001–2005. (a) shows the observed NDVI time series (squares), the resampled cubic spline-fitted data (crosses and line), the five year mean synoptic annual series (thick black line, displaced by −0.1 to ease viewing), and the Fourier fit (grey line), i.e. the sum of the annual, bi-annual and tri-annual harmonics of the TFA. Drop-out values are shown as −0.2. Details of the annual (solid line), bi-annual (dashed) and tri-annual (dotted) harmonics are shown in (b). The sum of these three harmonics is shown in grey for 1.5 years, as in (a). The horizontal line represents the overall mean and the vertical lines indicate the phase (timing) of the first peak of each of the Fourier harmonics in year 2001. The inset magnifies one year.

An example of the TFA of an NDVI time series for a single pixel in northern Europe is shown in Figure 5. The Fourier-fitted series, consisting of the summed annual, bi-annual and tri-annual harmonics, provided a good fit to the mean seasonal variation of the observed data (Figure 5a). The annual harmonic dominates the annual cycle of vegetation growth and has a large amplitude, indicating a major change between summer and winter NDVI values (Figure 5b). The second and third harmonics contribute less to the overall fit, but perform the important function of modulating the simple sinusoidal annual cycle. Figure 5b shows that the amplitude and phase of the tri-annual harmonic bring about a flattening and widening of the peak of the annual cycle, and thus improve the fit to the observed signal. The dominance of the annual cycle in Figure 5 is not surprising in a northern temperate habitat. Nearer the equator, and with other vegetation types, the bi-annual and tri-annual harmonics may modulate the annual cycle to a greater extent.

Global output layers of TFA for dLST and EVI for 2001–2005 are displayed in Figure 2 as three-channel colour composites, showing the mean, phase and amplitude of the first Fourier harmonic in the red, green and blue channels respectively. In Figure 2, areas in red indicate where the mean values are high and relatively constant throughout the year; those in bright green indicate where both mean and annual amplitude values are low and the peak values are reached later in the year; and those in bright blue indicate where the mean is relatively low, the peak occurs relatively early in the year and the annual amplitude of the signal is very pronounced. Areas in yellow ( = red+green), therefore, indicate high mean values and late peaks in the signal's annual cycle, and those in purple ( = red+blue) indicate high mean values and high annual amplitudes with early peaks. A little experience with this RGB colour scheme allows a direct, visual interpretation of habitat seasonality.

**Figure 6:**
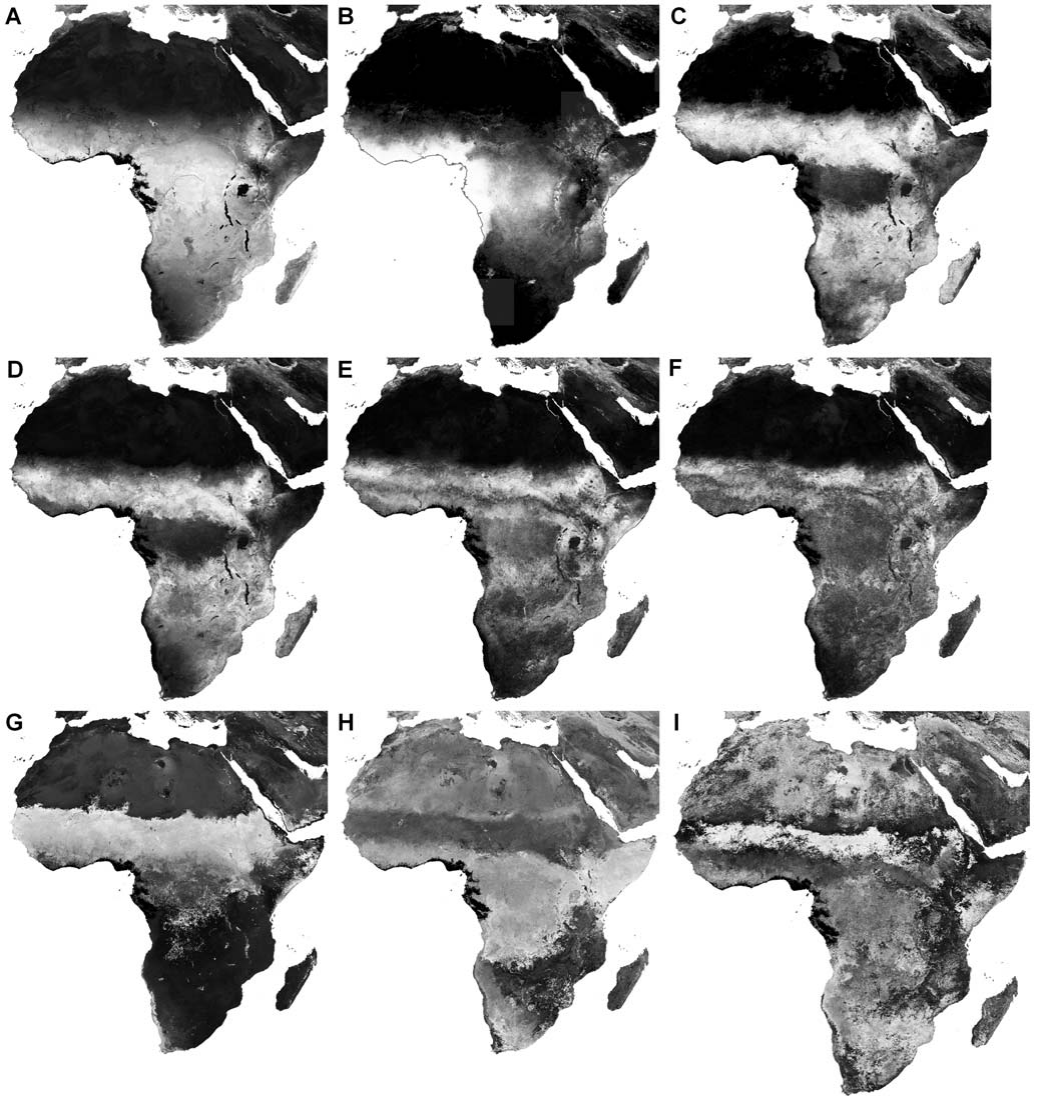
Selection of MODIS Enhanced Vegetation Index (EVI) temporal Fourier-processed output layers. Panels show: (a) mean, (b) the percentage of missing values in the time series and (c) the proportion of variance in the original time series described by annual, bi-annual, and tri-annual cycles combined. Amplitude of the (d) annual, (e) bi-annual, and (f) tri-annual cycle are shown in addition to the phase of the (g) annual , (h) bi-annual, and (i) tri-annual cycle in months. Data are histogram-equalized for display from minimum (black) to maximum value (white). A coastline was added to (b) to show the missing values (white) more clearly. Data are displayed in the MODIS sinusoidal projection.

To gain a more regional view, as well as display all Fourier harmonics, Figure 6 provides a selection of the 17 output layers for EVI across Africa. Specifications for all output layers for all products are given in Table S2.

Overall, the first three Fourier harmonics describe the observed seasonal pattern fairly well as shown by the percentage of variance explained (for MIR 55.7±23.9 [mean±SD], NDVI 58.8±32.1, EVI 51.9±30.6, dLST 83.1±21.0, nLST 79.1±24.7). The high level of variation around these global mean values of explained variance is often associated with habitat types. For example, deciduous woodland savannah vegetation shows a marked seasonality in NDVI and EVI, and a large proportion of the total variance of the signal is explained by the sum of the variances of the annual, bi-annual and tri-annual harmonics. Evergreen tropical rainforests on the other hand, do not show so much seasonality in the vegetation indices, and much of this variation appears to be noise; in these habitats the sum of the annual, bi-annual and tri-annual variances does not explain a large percentage of the (lower total) signal variance.

## Discussion

The processing chain presented here provides a powerful method for reliably and accurately capturing the synoptic seasonal dynamics of several environmental variables derived from the MODIS sensor. Major characteristics of the seasonality of each environmental variable can be described by only three Fourier harmonics involving seven variables (the mean, three amplitudes and three phases). Compared to the full time series, this represents a 16-fold reduction for reflectance-derived products collected at 16-day intervals (115 granules/7 output layers) and a 32-fold reduction for temperature products at 8-day intervals (230/7). These Fourier processed Terra MODIS data provide enhanced descriptions of the seasonality of natural environments at finer spatial and temporal resolution, compared with previous data sets (e.g. AVHRR) and thus should allow refined predictions of species' distributions.

### Critique of data and processing methodology

Although MODIS provides some of the finest spatial resolution multi-temporal global imagery available, several inescapable problems remain. The data are provided with a geolocational accuracy of 50 m (1σ) at nadir [44], but within a time series, pixels are viewed at different view angles with each repeat observation. The view angle determines the actual area of the Earth's surface observed by each pixel. Due to sensor geometry and the Earth's curvature, MODIS scans are elongated so that at the scan edge with high view zenith angles, the surface area actually viewed by one pixel is twice as long and 4.8 times wider than at nadir (the so-called “bow-tie” effect, [44]). Therefore each granule in a time series for a pixel relates to a different surface area on the Earth, depending on the view zenith angle (“pixel shift”, [51]). This might be overcome by excluding pixels with high view zenith angles during compositing or by aggregating pixels to coarser resolution [51].

Despite a high repeat frequency and a five year time series, insufficient reliable data were available to permit TFA in several cloudy or dark regions, e.g. the coastal regions of Nigeria and Cameroon in West Africa, the mountains of Venezuela, and many high latitude regions (see Figure 2, Figure 6b). Cloud contamination occurs at the same time each year in many of these regions, making elimination of such data gaps difficult. As longer time series become available, these gaps are more likely to be filled, although TFA will then first require some averaging across several years' of data.

Land surface temperatures, especially nLST, were noticeably lower over inland waters and their surrounding land pixels compared with more distant land pixels. This was due to cloud contamination above the inland waters, especially at night-time. The MODLAND land/water mask, which allows identification of inland and ephemeral water bodies, is therefore included within the data archive.

In addition to these geophysical constraints, there was a processing problem whereby the pixels in the westernmost column and northernmost row of each LST tile have lower values than the adjacent pixels, especially noticeable with nLST. This was due to simplifications in the MOD11A2 processing algorithm necessitated by processing limitations (Z. Wan, pers. comm.). Swath edges are clearly visible in the LST input and output layers. These problems are being resolved in future releases of the MOD11A2 data set (Z. Wan, pers. comm.).

The algorithm presented here first identified and then interpolated drop-out or suspect values, as these can affect the calculated TFA values if they are ignored rather than interpolated. Whilst other, non-linear interpolation methods may produce better TFA results than the linear interpolation used here, it was felt that the additional processing time required was not merited. Local, non-linear interpolation brings its own problems, e.g. choice of the range of data points over which to interpolate. TFA of the linearly interpolated results is itself a non-linear smoother of the entire time series, and so brings with it the advantages of global rather than local smoothing.

Although TFA as applied here provides a highly efficient data reduction method, capturing the seasonality of a time series for a synoptic year, it assumes that there were no longer-term cycles of any importance, or directional changes over time. Longer-term cycles may be captured by including multi-year Fourier harmonics in the output data sets, whilst trends may be captured by carrying out TFA over shorter periods of time, e.g. single year, 2 years, etc. and then looking for trends in the output layers (means, amplitudes and phases). However, whilst both longer-term cycles and trends can be important for investigating range changes and dynamics, the synoptic Fourier harmonics presented here should be sufficient for most species distribution modelling requirements that generally rely on historic data, often collected over long periods of time.

### Future

Longer time series of MODIS data offer several advantages. First, TFA of longer time series provides more representative measures of seasonality and allows assessment of change over time. Second, data gaps due to prolonged cloud contamination have a higher probability of being filled.

With the release of version 5 MODIS data sets, produced with improved processing algorithms, many of the problems highlighted above should be eliminated. Availability of finer spatial resolution (500 and 250 m) data sets will provide more locational detail. In the future, more accurate data sets will become available by combining data gathered by the Terra and Aqua satellites, both carrying the MODIS sensor. Other products, such as rainfall, vapour pressure deficit, evapotranspiration and snow cover are also suitable for TFA, although the abrupt nature of snow and rainfall (close to zero for some of the year, with intense periods at other times) may require more than three harmonics to fit the time series satisfactorily.

Whilst the Terra satellite is already beyond its originally scheduled operational lifespan, NASA appears to be committed to maintain the supply of MODIS data until the sensor becomes non-operational or until *c*. 2013 when the Visible/Infrared Imager/Radiometer Suite (VIIRS) sensor is scheduled to begin acquiring data. Barring accidents or unexpected equipment failures, it is thought that the MODIS sensor might continue working for another *c*. 6 years.

These global temporal Fourier processed MODIS data layers for 2001–2005 represent a new and valuable resource for the scientific community, and are available to collaborators upon request.

## Supporting Information

Table S1 Geo-referencing information for global MODIS data.
(0.04 MB DOC)

Table S2 Description of Temporal Fourier Analysis output layers. The table gives details of scaling factors to be applied to the data (i.e. the digital numbers, x, stored in the files), the resulting data units and observed geophysical ranges. The minimum (mn) and maximum (mx) layers are derived from the TFA fit to the data and may therefore occasionally exceed the possible geophysical limits. In the absence of data drop-outs, the mean (a0) would also be the arithmetic mean of the input data; in practice the TFA mean is the arithmetic mean of the interpolated satellite data to which the final Fourier fit is made.
(0.22 MB DOC)
