# Supplementary material for: Global Data for Ecology and Epidemiology: A Novel Algorithm for Temporal Fourier Processing MODIS Data
Source: PLoS One. 2008 Jan 9;3(1):e1408. doi: 10.1371/journal.pone.0001408 (PMC2171368; doi:10.1371/journal.pone.0001408)
Supplement: Table S1 — Geo-referencing information for global MODIS data. (0.04 MB DOC) [file pone.0001408.s001.doc]

**Table S1. Geo-referencing information for global MODIS data.**

| Details | Projection parameter |
| --- | --- |
| Projection | sinusoidal |
| Image size, columns | 43200 |
| Image size, rows | 21600 |
| Upper-left X coordinate, pixel centre | -20014646.041 m |
| Upper-left Y coordinate, pixel centre | 10007091.364 m |
| Units | meters |
| Pixel size, x | 926.62543305 m |
| Pixel size, y | 926.62543305 m |
| Spheroid | MODIS specific |
| Radius of sphere | 6371007.181 m |
| Central meridian | 0° 0’ 0.00” |
| Easting and Northing | 0 m |
