# Supplementary material for: Global Data for Ecology and Epidemiology: A Novel Algorithm for Temporal Fourier Processing MODIS Data
Source: PLoS One. 2008 Jan 9;3(1):e1408. doi: 10.1371/journal.pone.0001408 (PMC2171368; doi:10.1371/journal.pone.0001408)
Supplement: Table S2 — Description of Temporal Fourier Analysis output layers. The table gives details of scaling factors to be applied to the data (i.e. the digital numbers, x, stored in the files), the resulting data units and observed geophysical ranges. The minimum (mn) and maximum (mx) layers are derived from the TFA fit to the data and may therefore occasionally exceed the possible geophysical limits. In the absence of data drop-outs, the mean (a0) would also be the arithmetic mean of the input data; in practice the TFA mean is the arithmetic mean of the interpolated satellite data to which the final Fourier fit is made. (0.22 MB DOC) [file pone.0001408.s002.doc]

**Table S2. Description of Temporal Fourier Analysis output layers.**

| File Name | Layer type | Layer description | Scaling | Units | Geophysical min. | Geophysical max. |
| --- | --- | --- | --- | --- | --- | --- |
| ws1503a0 | TFA | Middle Infrared (MIR) mean | *x* / 10000 | No units† | 0 | 0.759 |
| ws1503a1 | TFA | MIR annual amplitude | *x* / 10000 | No units† | 0 | 0.326 |
| ws1503a2 | TFA | MIR bi-annual amplitude | x / 10000 | No units† | 0 | 0.142 |
| ws1503a3 | TFA | MIR tri-annual amplitude | *x* / 10000 | No units† | 0 | 0.073 |
| ws1503p1 | TFA | MIR phase of annual cycle | *x* / 100 | months | 0 | 12 |
| ws1503p2 | TFA | MIR phase of bi-annual cycle | *x* / 100 | months | 0 | 6 |
| ws1503p3 | TFA | MIR phase of tri-annual cycle | *x* / 100 | months | 0 | 4 |
| ws1503mn | TFA | minimum MIR | *x* / 10000 | No units† | -0.0279 | 0.747 |
| ws1503m*x* | TFA | maximum MIR | *x* / 10000 | No units† | 0 | 0.828 |
| ws1503vr | TFA | MIR variance | *x* / 10000 | No units† | 0 | 0.059 |
| ws1503d1 | TFA | MIR pvs described by annual cycle | *x* | % | 0 | 99 |
| ws1503d2 | TFA | MIR pvs described by bi-annual cycle | *x* | % | 0 | 87 |
| ws1503d3 | TFA | MIR pvs described by tri-annual cycle | *x* | % | 0 | 62 |
| ws1503da | TFA | MIR pvs described by annual, bi- and tri-annual cycles | *x* | % | 0 | 100 |
| ws1503e1 | TFA | Percentage missing values in MIR time series | *x* | % | 0 | 100 |
| ws1503e2 | TFA | Percentage of values outside MIR geophysical limits | *x* | % | 0 | 80 |
| ws1503e3 | TFA | Percentage of Fourier fit values departing from initially interpolated MIR values. | *x* | % | 0 | 97 |
|  |  |  |  |  |  |  |
| ws1507a0 | TFA | daytime Land Surface Temperature (dLST) mean | *x* / 50 | °K | 0 | 328.28 |
| ws1507a1 | TFA | dLST annual amplitude | *x* / 50 | °K | 0 | 39.84 |
| ws1507a2 | TFA | dLST bi-annual amplitude | *x* / 50 | °K | 0 | 10.94 |
| ws1507a3 | TFA | dLST tri-annual amplitude | *x* / 50 | °K | 0 | 6.32 |
| ws1507p1 | TFA | dLST phase of annual cycle | *x* / 100 | months | 0 | 12 |
| ws1507p2 | TFA | dLST phase of bi-annual cycle | *x* / 100 | months | 0 | 6 |
| ws1507p3 | TFA | dLST phase of tri-annual cycle | *x* / 100 | months | 0 | 4 |
| ws1507mn | TFA | minimum dLST | *x* / 50 | °K | 0 | 325.28 |
| ws1507mx | TFA | maximum dLST | *x* / 50 | °K | 0 | 334.16 |
| ws1507vr | TFA | dLST variance | *x* | °K^2^ | 0 | 817 |
| ws1507d1 | TFA | dLST pvs described by annual cycle | *x* | % | 0 | 99 |
| ws1507d2 | TFA | dLST pvs described by bi-annual cycle | *x* | % | 0 | 80 |
| ws1507d3 | TFA | dLST pvs described by tri-annual cycle | *x* | % | 0 | 47 |
| ws1507da | TFA | dLST pvs described by annual, bi- and tri-annual cycles | *x* | % | 0 | 100 |
| ws1507e1 | TFA | Percentage missing values in dLST time series | *x* | % | 0 | 100 |
| ws1507e2 | TFA | Percentage of values outside dLST geophysical limits | *x* | % | 0 | 80 |
| ws1507e3 | TFA | Percentage of Fourier fit values departing from initially interpolated dLST values | *x* | % | 0 | 95 |
|  |  |  |  |  |  |  |
| ws1508a0 | TFA | night-time Land Surface Temperature (nLST) mean | *x* / 50 | °K | 0 | 306.02 |
| ws1508a1 | TFA | nLST annual amplitude | *x* / 50 | °K | 0 | 31.4 |
| ws1508a2 | TFA | nLST bi-annual amplitude | *x* / 50 | °K | 0 | 10.82 |
| ws1508a3 | TFA | nLST tri-annual amplitude | *x* / 50 | °K | 0 | 6.92 |
| ws1508p1 | TFA | nLST phase of annual cycle | *x* / 100 | months | 0 | 12 |
| ws1508p2 | TFA | nLST phase of bi-annual cycle | *x* / 100 | months | 0 | 6.01 |
| ws1508p3 | TFA | nLST phase of tri-annual cycle | *x* / 100 | months | 0 | 4 |
| ws1508mn | TFA | minimum nLST | *x* / 50 | °K | 0 | 302.8 |
| ws1508mx | TFA | maximum nLST | *x* / 50 | °K | 0 | 310.66 |
| ws1508vr | TFA | nLST variance | *x* | °K^2^ | 0 | 503 |
| ws1508d1 | TFA | nLST pvs described by annual cycle | *X* | % | 0 | 91 |
| ws1508d2 | TFA | nLST pvs described by bi-annual cycle | *X* | % | 0 | 82 |
| ws1508d3 | TFA | nLST pvs described by tri-annual cycle | *X* | % | 0 | 66 |
| ws1508da | TFA | nLST pvs described by annual, bi- and tri-annual cycles | *x* | % | 0 | 100 |
| ws1508e1 | TFA | Percentage missing values in nLST time series | *x* | % | 0 | 100 |
| ws1508e2 | TFA | Percentage of values outside nLST geophysical limits | *x* | % | 0 | 81 |
| ws1508e3 | TFA | Percentage of Fourier fit values departing from initially interpolated nLST values | *x* | % | 0 | 95 |
|  |  |  |  |  |  |  |
| ws1514a0 | TFA | Normalised Difference Vegetation Index (NDVI) mean | (*x* / 1000) - 1 | No units† | -0.2 | 0.995 |
| ws1514a1 | TFA | NDVI annual amplitude | (*x* / 1000) | No units† | -0.2 | 0.605 |
| ws1514a2 | TFA | NDVI bi-annual amplitude | (*x* / 1000) | No units† | -0.2 | 0.387 |
| ws1514a3 | TFA | NDVI tri-annual amplitude | (*x* / 1000) | No units† | -0.2 | 0.241 |
| ws1514p1 | TFA | NDVI phase of annual cycle | *x* / 100 | months | 0 | 12 |
| ws1514p2 | TFA | NDVI phase of bi-annual cycle | *x* / 100 | months | 0 | 6 |
| ws1514p3 | TFA | NDVI phase of tri-annual cycle | *x* / 100 | months | 0 | 4 |
| ws1514mn | TFA | minimum NDVI | (*x* / 1000) - 1 | No units† | 0 | 0.993 |
| ws1514mx | TFA | maximum NDVI | (*x* / 1000) - 1 | No units† | 0 | 1.05 |
| ws1514vr | TFA | NDVI variance | *x* / 10000 | No units† | 0 | 0.211 |
| ws1514d1 | TFA | NDVI pvs described by annual cycle | *x* | % | 0 | 98 |
| ws1514d2 | TFA | NDVI pvs described by bi-annual cycle | *x* | % | 0 | 91 |
| ws1514d3 | TFA | NDVI pvs described by tri-annual cycle | *x* | % | 0 | 78 |
| ws1514da | TFA | NDVI pvs described by annual, bi- and tri-annual cycles | *x* | % | 0 | 100 |
| ws1514e1 | TFA | Percentage missing values in NDVI time series | *x* | % | 0 | 100 |
| ws1514e2 | TFA | Percentage of values outside NDVI geophysical limits | *x* | % | 0 | 100 |
| ws1514e3 | TFA | Percentage of Fourier fit values departing from initially interpolated NDVI values | *x* | % | 0 | 99 |
|  |  |  |  |  |  |  |
| ws1515a0 | TFA | Enhanced Vegetation Index (EVI) mean | (*x* / 1000) - 1 | No units† | -0.2 | 0.852 |
| ws1515a1 | TFA | EVI annual amplitude | (*x* / 1000) | No units† | -0.2 | 0.487 |
| ws1515a2 | TFA | EVI bi-annual amplitude | (*x* / 1000) | No units† | -0.2 | 0.347 |
| ws1515a3 | TFA | EVI tri-annual amplitude | (*x* / 1000) | No units† | -0.2 | 0.245 |
| ws1515p1 | TFA | EVI phase of annual cycle | *x* / 100 | Months | 0 | 12 |
| ws1515p2 | TFA | EVI phase of bi-annual cycle | *x* / 100 | Months | 0 | 6 |
| ws1515p3 | TFA | EVI phase of tri-annual cycle | *x* / 100 | Months | 0 | 4 |
| ws1515mn | TFA | minimum EVI | (*x* / 1000) - 1 | No units† | 0 | 0.804 |
| ws1515mx | TFA | maximum EVI | (*x* / 1000) - 1 | No units† | 0 | 0.957 |
| ws1515vr | TFA | EVI variance | *x* / 10000 | No units† | 0 | 0.140 |
| ws1515d1 | TFA | EVI pvs described by annual cycle | *x* | % | 0 | 98 |
| ws1515d2 | TFA | EVI pvs described by bi-annual cycle | *x* | % | 0 | 94 |
| ws1515d3 | TFA | EVI pvs described by tri-annual cycle | *x* | % | 0 | 84 |
| ws1515da | TFA | EVI pvs described by annual, bi- and tri-annual cycles | *x* | % | 0 | 100 |
| ws1515e1 | TFA | Percentage missing values in EVI time series | *x* | % | 0 | 100 |
| ws1515e2 | TFA | Percentage of values outside EVI geophysical limits | *x* | % | 0 | 100 |
| ws1515e3 | TFA | Percentage of Fourier fit values departing from initially interpolated EVI values | *x* | % | 0 | 91 |
|  |  |  |  |  |  |  |
| wsv490lw | MSK | MODLAND v4 land water mask  (1-land, 2-shoreline, 3-inland water, 4-ephemeral water) | *x* | No units# | 1 | 4 |
| wsv490wa | MSK | MODLAND v4 water mask (as wsv490lw) | *x* | No units# | 2 | 4 |
| wsv4mask | MSK | MODLAND v4 land mask (1-land + shoreline) | *x* | No units# | 0 | 1 |

The table gives details of scaling factors to be applied to the data (i.e. the digital numbers, *x*, stored in the files), the resulting data units and observed geophysical ranges. The minimum (mn) and maximum (mx) layers are derived from the TFA fit to the data and may therefore occasionally exceed the possible geophysical limits. In the absence of data drop-outs, the mean (a0) would also be the arithmetic mean of the input data; in practice the TFA mean is the arithmetic mean of the interpolated satellite data to which the final Fourier fit is made.

TFA - Temporal Fourier analysis, MSK - mask layers; pvs - proportion of variance of the original signal, † MIR, NDVI and EVI are dimensionless ratios, # categorical data. Geophysical minimum and geophysical maximum values do not include masks.
